# Supplementary material for: Patient participant, healthcare professional, and stakeholder perspectives on the Pharmacy Homeless Outreach Engagement Non-medical Independent prescribing Rx (PHOENIx) community pharmacy pilot randomised controlled trial
Source: PLoS One. 2025 Jun 27;20(6):e0324654. doi: 10.1371/journal.pone.0324654 (PMC12204468; doi:10.1371/journal.pone.0324654)
Supplement: Attachment — Submitted filename: Response to Reviewers.docx [file pone.0324654.s002.docx]

Dear Editor/ Reviewer,

Thank you for your considered and helpful comments on the manuscript entitled: ‘Patient participant, healthcare professional, and stakeholder perspectives on the Pharmacy Homeless Outreach Engagement Non-medical Independent prescribing Rx (PHOENIx) community pharmacy pilot randomised controlled trial’.

The authors have reviewed the comments provided and have made amendments where appropriate. A table of responses to comments can be found below:

| Table of reviewer comments and response from authors | | |
| --- | --- | --- |
| **Reviewer** | **Comment** | **Response** |
| 1 | There are few repetitive content in the introduction and results sections. You may try to avoid that to avoid distracting the reader of your manuscript. | The text was checked for clarity and consistency. |
| 1 | Please emphasize the unique contributions of your study, particularly its implications for scaling the PHOENIx model. | Text added to the discussion that aims to highlight the unique contribution to the field of study, as well as implications/ suggestions for scalability. |
| 1 | In your conclusion, you may consider discussing how findings can guide interventions for other underserved populations beyond PEH. | Text has been added to the conclusion to highlight how successful engagement of an underserved population (PEH) can be used as a guide for future interventions looking to take a person-centred approach with other marginalised groups e.g. migrant population. |
| 2 | What percentage of PEH represent the sample size for the cities of Glasgow and Birmingham? | This has been noted, with appropriate text added to ‘Considerations for Future RCT’ section within the discussion. |
| 2 | The authors need to address in the discussion section what strategies they will use to increase the number of PEH participants for future studies? | This has been noted, with appropriate text added to ‘Considerations for Future RCT’ section within the discussion. This includes suggestions for improving recruitment and retention of study participants. |

Other changes that have been made to the manuscript include:

- Amended formatting to meet PLOS One submission guidelines, including correct heading formatting.
- An addition of two further references, added in response to reviewer twos request for figures relating to percentage of number of PEH in Glasgow and Birmingham.
- All references have been checked, and there are no articles that have been retracted at this time.

In relation to the data availability statement, as highlighted by the editor, we would ask to request an exemption on the grounds that by making the data available we would be in breach of our ethical approval granted by NHS (REC reference 22/EM/0119). The ethics application, and therefore participant consent, state that anonymised data might be shared with qualified researchers but does not specify being made publicly available. As a result, the data cannot be made publicly available at this time. If required, the approved ethics application can be provided.

Best wishes,

Hannah Scobie
